# Delimitation of lymphatic filariasis transmission risk areas: a geo-environmental approach

**DOI:** 10.1186/1475-2883-5-12

**Published:** 2006-11-09

**Authors:** Shanmugavelu Sabesan, Hari Kishan K Raju, AdiNarayanan Srividya, Pradeep Kumar Das

**Affiliations:** 1 Vector Control Research Centre, Medical Complex, Indira Nagar, Pondicherry – 605 006, India

## Abstract

**Background:**

The Global Programme to Eliminate Lymphatic Filariasis (GPELF) depends upon Mass Drug Administration (MDA) to interrupt transmission. Therefore, delimitation of transmission risk areas is an important step, and hence we attempted to define a geo-environmental risk model (GERM) for determining the areas of potential transmission of lymphatic filariasis.

**Methods:**

A range of geo-environmental variables has been selected, and customized on GIS platform to develop GERM for identifying the areas of filariasis transmission in terms of "risk" and "non-risk". The model was validated through a 'ground truth study' following standard procedure using GIS tools for sampling and Immuno-chromotographic Test (ICT) for screening the individuals.

**Results:**

A map for filariasis transmission was created and stratified into different spatial entities, "risk' and "non-risk", depending on Filariasis Transmission Risk Index (FTRI). The model estimation corroborated well with the ground (observed) data.

**Conclusion:**

The geo-environmental risk model developed on GIS platform is useful for spatial delimitation purpose on a macro scale.

## Background

Lymphatic filariasis (LF), a mosquito vector-borne disease is a major public health problem in many parts of the tropics. International Task Force for Disease Eradication has identified the LF, as one of the six diseases considered eradicable or potentially eradicable [1]. For countries with national programmes [2] an important requirement is to have information on spatial spread and status of the risk of filarial infection, which would facilitate an appropriate planning for control/elimination.

Lymphatic filariasis distribution map has been created for India, based on the historical data available in 2000 [3]. Further, it was realized that the traditional method for delimitation of areas, using the night blood examination survey is useful only for identifying the areas of risk at the micro level. This method is time consuming, relatively insensitive, labour intensive and the results are influenced by human factors. Spatial interpolation of this data on a macro scale is not possible, since the processes underlying the pattern of filariasis distribution are governed by environmental conditions [4]. This implies that the risk of filariasis can only be defined in a geographical area, as there is local variation in the environmental conditions. With this background, a geo-environmental risk map has been created for Tamil Nadu region of southern India, to delimit the areas, indicating the potential risk of transmission of lymphatic filariasis.

## Methods

### Study area

Tamil Nadu region of southern India represents a wide range of geo-environmental features. The study area (1,30,058 sq. km) lies between 8° 27' 2" N – 18° 20' 55" N and 74° 16' 17" E – 80° 11' 45"E. This area receives monsoon rainfall, i.e., South-West (June – Sep.) and North-East (Oct. – Dec.). The rainfall is moderate to high, with an annual average of 800 mm to 3000 mm. The mean annual temperature is 25°C – 27.5°C. Altitude ranges, sea level to 1800 m. Major soil types include: (i) Red sandy soil, (ii) Red loamy soil, (iii) Red and mixed black soil, (iv) Black soil and (v) Alluvial soil. The total population of the study area is 56.3 million (Source: Census of India 2001). The population density ranges from 106 to 3917 persons per sq. km.

### GIS data base construction

District level digital map of Tamil Nadu (Survey of India administrative boundary maps – Scale 1:50,000) was used. All GIS database are developed, using ArcView 3.2, ArcGIS 8 software (ESRI, Redlands, CA) and ERDAS IMAGINE 8 (ERDAS, Atlanta, GA), an Image processing software.

### Geo-environmental and climatological data source

The possible geo-environmental risk factors that are likely to influence the occurrence of lymphatic filariasis were identified. Their role (either directly or indirectly) in the transmission of filariasis was accessed [5]. Accordingly, the Geo-environmental factors viz. Altitude, Temperature, Rainfall, Relative Humidity, Soil Type and the Land Use/Land cover (Level – I category) were considered for this study. Data on soil and agro-ecological features were obtained from the National Bureau of Soil and Land Use Planning, Nagpur. The detailed weather data was received from the Indian Meteorological Department, Pune.

### Geo-Environmental Risk Model (GERM)

Variables such as altitude, temperature, rainfall, relative humidity, soil type and land use/cover either individually or in combination are known to be associated with the occurrence of filariasis [5,6]. We have identified the range of values of these variables that are reportedly conducive for the transmission of filariasis [5]. With the exception of land use/cover profile, all the other variables were found to be associated (based on multivariate analysis – done separately: unpublished) with the occurrence of filariasis in the study area and these variables were used for computing a composite index, to create a filariasis transmission risk index (FTRI) for developing the model.

The model customized the environmental parameters, encompassing the ranges of different variables associated with transmission of filariasis as: altitude 0 – 1800 m above sea level, a mean temperature range of 8°C – 37°C, annual rainfall from 300 mm – 1500 mm and relative humidity 40% – 90%, in addition to the different soil types for deriving the FTRI. To accomplish this, each value of these geo-environmental variables was given scores (in the range/characteristics provided above) for different geographical areas. Summing up the scores of these geo-environmental variables was undertaken for a particular area, i.e., given mathematically as follows:

FTRIforanarea =∑i=15Yi
 MathType@MTEF@5@5@+=feaafiart1ev1aaatCvAUfKttLearuWrP9MDH5MBPbIqV92AaeXatLxBI9gBaebbnrfifHhDYfgasaacH8akY=wiFfYdH8Gipec8Eeeu0xXdbba9frFj0=OqFfea0dXdd9vqai=hGuQ8kuc9pgc9s8qqaq=dirpe0xb9q8qiLsFr0=vr0=vr0dc8meaabaqaciaacaGaaeqabaqabeGadaaakeaaieaacqWFgbGrcqWFubavcqWFsbGucqWFjbqscqWFGaaicqWFMbGzcqWFVbWBcqWFYbGCcqWFGaaicqWFHbqycqWFUbGBcqWFGaaicqWFHbqycqWFYbGCcqWFLbqzcqWFHbqycqqGGaaicqGH9aqpdaaeWbqaaiabdMfazjabdMgaPbWcbaGaemyAaKMaeyypa0JaeGymaedabaGaeGynaudaniabggHiLdaaaa@4A7B@

Where ∑*Yi *= Y_1_+Y_2_+Y_3_+Y_4_+Y_5_

Y_1_, Y_2_, Y_3_, Y_4 _and Y_5 _are scores for Altitude, Temperature, Relative Humidity, Rainfall and Soil type respectively.

In order to indicate the FTRI for a locality as a standardized quantity, it is represented in terms of percentage as follows:

FTRI of a locality= ∑i=15Yi of a localityMaximum value of ∑i−15Yi in the study area×100
 MathType@MTEF@5@5@+=feaafiart1ev1aaatCvAUfKttLearuWrP9MDH5MBPbIqV92AaeXatLxBI9gBaebbnrfifHhDYfgasaacH8akY=wiFfYdH8Gipec8Eeeu0xXdbba9frFj0=OqFfea0dXdd9vqai=hGuQ8kuc9pgc9s8qqaq=dirpe0xb9q8qiLsFr0=vr0=vr0dc8meaabaqaciaacaGaaeqabaqabeGadaaakeaacqqGgbGrcqqGubavcqqGsbGucqqGjbqscqqGGaaicqqGVbWBcqqGMbGzcqqGGaaicqqGHbqycqqGGaaicqqGSbaBcqqGVbWBcqqGJbWycqqGHbqycqqGSbaBcqqGPbqAcqqG0baDcqqG5bqEcqGH9aqpcqqGGaaidaWcaaqaamaaqahabaGaemywaKLaemyAaKgaleaacqWGPbqAcqGH9aqpcqaIXaqmaeaacqaI1aqna0GaeyyeIuoakiabbccaGiabd+gaVjabdAgaMjabbccaGiabdggaHjabbccaGiabdYgaSjabd+gaVjabdogaJjabdggaHjabdYgaSjabdMgaPjabdsha0jabdMha5bqaaiabd2eanjabdggaHjabdIha4jabdMgaPjabd2gaTjabdwha1jabd2gaTjabbccaGiabdAha2jabdggaHjabdYgaSjabdwha1jabdwgaLjabbccaGiabd+gaVjabdAgaMjabbccaGmaaqahabaGaemywaKLaemyAaKMaeeiiaaIaemyAaKMaemOBa4MaeeiiaaIaemiDaqNaemiAaGMaemyzauMaeeiiaaIaem4CamNaemiDaqNaemyDauNaemizaqMaemyEaKNaeeiiaaIaemyyaeMaemOCaiNaemyzauMaemyyaegaleaacqWGPbqAcqGHsislcqaIXaqmaeaacqaI1aqna0GaeyyeIuoaaaGccqGHxdaTcqaIXaqmcqaIWaamcqaIWaamaaa@98C6@

This approach was used to identify the areas to assess the potential for risk of filariasis transmission in Tamil Nadu, India.

### Validation of GERM

#### Sampling design

##### Grid sampling

Throughout the Tamil Nadu region, where the model was developed, an area of 100 × 350 km was selected representing all geo-environmental features for validation purposes. The region was divided into 10 × 10 km grids, so that each grid would contain at least one village (rural)/ward (urban). In the non-risk zone, all the villages/wards falling on either intersection points or nearer to them were selected for the survey, since the negative estimation in the model was more critical from the application point of view. Thus, a total of 35 geo-coded sites (intersection points) were required to be surveyed in the "non-risk" zone.

In the rest of the areas ("risk" zone), 10% of the total intersection points (villages/wards) were selected on a systematic random basis, to minimize the cost of survey but at the same time ensured a fair spatial representation of the area. The number of sites thus selected for the survey in the risk area was 25. A total of sixty sites were surveyed from the study area.

##### Screening the children, using ICT kits

In all the selected sites, children below 15 years of age were screened for antigenaemia positivity for *Wuchereria bancrofti *(Ag), using the Immuno-Chromotographic Test (ICT). The likelihood of positive in this group was higher, besides indicating the locally acquired infection or indigenous transmission.

##### Sample size

Assuming a minimum expected 1% antigenaemia prevalence in the age class < 15 years and allowing an error of 2.5%, the required sample size was estimated to be 58 in a population of about 1500 in a village. Therefore, 60 children were screened in each site. This was achieved by visiting either all or selected sections of the schools, located in these sites (villages/wards), following a systematic random procedure. Ethical clearance was obtained from the Institutional Ethics Committee. The children were screened only with the informed consent of their guardian.

### Statistical analysis

The relationship between the FTRI and the filarial antigenaemia in the study sites was analyzed, using logistic regression (with FTRI as the independent variable, and filarial antigenaemia prevalence as the dependent variable). The dependent variable is binary in nature indicating the presence or absence of filariasis transmission in the area.

## Results

**Figure 1:**
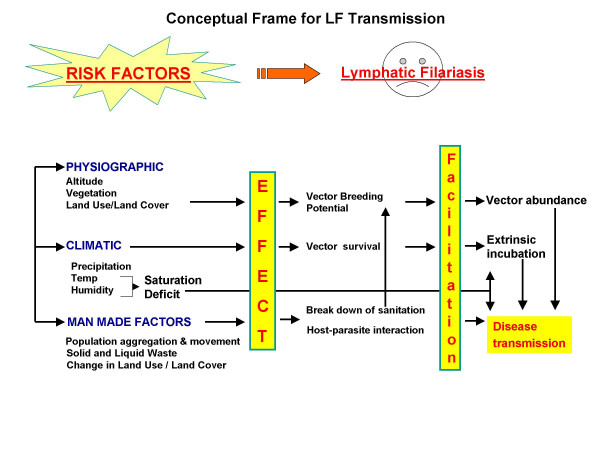
Conceptual Frame for LF Transmission.

A conceptual frame for lymphatic filariasis transmission developed on the basis of current knowledge is shown in Fig. 1. Filariasis transmission like other vector-borne infectious diseases is dependent on the geo-environmental variables (physiographic & climatic) at a macro level. Physiographic factors mainly contributed to vector abundance; whereas the climatic factors influenced the extrinsic incubation (of parasites) directly, as well as via vector survival. Once the environmental conditions are conducive, the human (demographic) factors become the determinants contributing to the occurrence of filariasis at the micro level.

**Figure 2:**
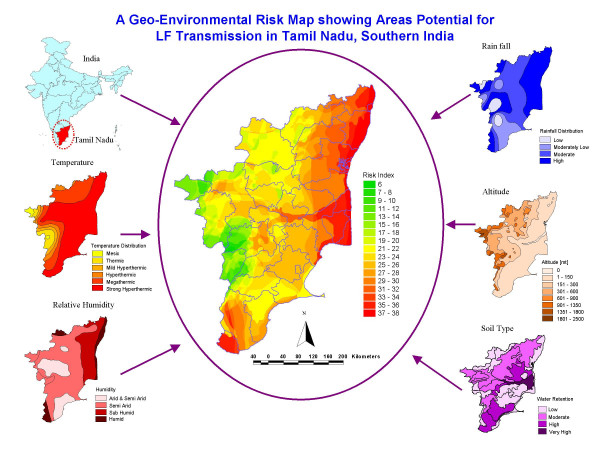
A geo-environmental risk map showing areas potential for LF transmission in Tamil Nadu, Southern India.

Based on the relative contribution of geo-environmental factors, a filariasis transmission risk model was developed and map generated (Fig. 2) on a GIS platform, using GERM. Depending on the FTRI values, the map was stratified into different spatial entities, from high "risk" ('red') to "non-risk" ('green'). The derived FTRI values ranged from 6 to 38 in the study sites. As shown in the map (Fig. 2), there is no risk of transmission where the FTRI value < 16. The risk of transmission begins with FTRI value 17, and the potential risk increases with increasing value of FTRI.

### Model validation

A total of 3600 children were examined for presence of filarial antigenaemia from the selected 60 sites. The results of the survey are given in Table 1.

**Table 1:** 

**Filariasis Risk status of the area**	**No. of sites selected**	**No. of sites detected positive by the ICT test**	**No. of individuals tested**		
			
			**Positive**	**Negative**	**Total**
"Non-risk"	35	0	0	2100	2100
"Risk"	25	8	28	1472	1500
Total	60	8	28	3572	3600

None of the 2100 children screened from "non-risk" area were positive for filarial antigenemia. Whilst the 28 positive children recorded were from the "risk" area. Based on the antigenaemia (ICT data) positivity/negativity of sites, the sensitivity of the model was found to be 100% (95% limits: 59.8% – 100.0%) and specificity 67.3% (52.8% – 79.3%). The positive and negative expected values were being 32% (15.7 – 53.6) and 100% respectively.

The relationship between the FTRI and the prevalence of filarial antigenaemia has shown that the model is fitted (93.3%) the data well (Hosmer & Lemeshow goodness of fit test: χ^2 ^= 2.27, D.F. = 6, P = 0.892), thereby demonstrating that the FTRI was a significant indicator of "non-risk" status of filariasis in an area.

## Discussion

The geo-environmental risk model developed on a GIS platform could be employed for spatial delimitation of filariasis risk on a macro scale, particularly to identify "non-risk" areas, more precisely. *W. bancrofti *transmission is largely determined by the geo-environmental variables, and hence it is possible to identify the areas where risk of transmission can be determined on a macro-scale. Environmental conditions are widely conducive, to transmission efficiency; human factors are also key determinants contributing to the local occurrence of filariasis.

The influence of human factors such as population density, movement, economic status, occupation, literacy level and health seeking behaviour on the occurrence of lymphatic filariasis at micro level has been described elsewhere [7,8]. Similarly, the vector abundance may vary widely at micro level depending on the geo-physical and human associated factors, but the 'vectorial capacity' (vector survival and capacity for parasite development) and the transmission of infection are greatly determined by the geo-environmental factors at macro level. Further, the implementation of mass drug administration (MDA) using diethyl carbamazine citrate (DEC), and albendazole towards the elimination of LF in several parts of India, including Tamil Nadu region in the recent years (3 – 5 years in different administrative units) is expected to reduce community infection intensity. However, our model does not aim to estimate the prevalence of cases, but only to identify the areas potential for risk of LF transmission at macro level.

Hassan *et al*.,[9] predicted villages at risk for filariasis in the Nile Delta using remote sensing (RS) and geographic information system (GIS) technologies. They used Landsat Thematic Mapper (TM) data to generate a map of land cover as well as spectral indices such as Normalized Difference Vegetation Index (NDVI) and moisture index. Discriminant functions generated for these variables were able to correctly predict 80% and 74% of high and low prevalence villages, respectively, with an overall accuracy of 77%. Lindsay and Thomas [10] hypothesized that the distribution of lymphatic filariasis is governed by climate. The climate at sites in Africa where surveys for lymphatic filariasis had taken place was characterized using computerized climate surfaces. Logistic regression analysis of the climate variables predicted with 76% accuracy whether sites had microfilaraemic patients or not. A similar procedure was used to map risk of microfilaraemia in Egypt [10], where the dominant vector species differs from those in sub – Saharan Africa, and thereby judged incorrectly. By overlaying risk maps on a 1990 population grid, and adjusting for recent population increases, they estimated that around 420 million people would be exposed to this infection in Africa in the year 2000. Here, parasite distribution has been predicted by relatively straightforward statistical methods that give correlations between environmental factors and parasite distribution.

In the present study, each of the geo-environmental factors was not taken as a separate entity, but all of them have been considered together a composite index (FTRI), since a combination of factors are responsible for transmission [5]. The results obtained now indicate that the areas with "non-risk" of filariasis transmission can be identified at macro level using the GERM, particularly for a situation like in India, where some geographical areas are not surveyed even one time, and their endemicity not known. However, through this model, it may not be possible to identify the positive cases from the potential "risk" areas (at 'micro' level) with any of the existing tools. Also, it may be surmised that all the people live at risk of infection need not be 'infective'. But, one can expect very well a relatively high risk of LF transmission with the increasing values of FTRI in the "risk" areas.

## Conclusion

It has been demonstrated in the present model that no transmission takes place in any part of the "non-risk" areas. Since the 'negative' determination of this model is excellent, all the "non-risk" areas could straightaway be omitted from covering the LF elimination under the MDA programme. Further, it is foreseeable that this approach could be a method of choice for filariasis delimitation for a country having a vast geographical area, in view of its rapidity and reliability.

## Authors' contributions

All authors have contributed significantly to the work. SS had a substantial contribution to the conception, design and preparation of the manuscript. KHKR: acquisition of data and facilitated model development. AS: analyzed the data and its interpretation. PKD: gave a critical review with intellectual input in conception and design.

## Competing interests

The author(s) declare that they have no competing interests.
